# Photoconductivity of Thin Films Obtained from a New Type of Polyindole

**DOI:** 10.3390/ma15010228

**Published:** 2021-12-29

**Authors:** Renat B. Salikhov, Akhat G. Mustafin, Ilnur N. Mullagaliev, Timur R. Salikhov, Anastasiia N. Andriianova, Lyaysan R. Latypova, Ildus F. Sharafullin

**Affiliations:** 1 Institute of Physics and Technology, Bashkir State University, Zaki Validi St. 32, 450076 Ufa, Russia; ilnur9409@mail.ru (I.N.M.); timur-salikhov@yandex.ru (T.R.S.); sharafullinif@yandex.ru (I.F.S.); 2 Faculty of Chemistry, Bashkir State University, Zaki Validi St. 32, 450076 Ufa, Russia; agmustafin@gmail.com; 3 Ufa Institute of Chemistry of the Russian Academy of Sciences, Pr. Oktyabrya 71, 450054 Ufa, Russia; anastasia.shishkina1993@mail.ru (A.N.A.); lesa06091991@yandex.ru (L.R.L.)

**Keywords:** polymer, polyindole, photoconductivity, band gap, phototransistor, mobility

## Abstract

The optoelectronic properties of a new poly(2-ethyl-3-methylindole) (MPIn) are discussed in this paper. The absorption and photoluminescence spectra were studied. The electronic spectrum of MPIn showed a single absorption maximum at 269 nm that is characteristic of the entire series of polyindoles. The fluorescence spectra show that the emission peaks of the test sample are centered around 520 nm. The photoconductivity of thin film samples of MPIn polyindole was studied by measuring the current-voltage characteristics under ultraviolet radiation with a wavelength of 350 nm. Samples of phototransistors were obtained, where thin films of MPIn polyindole were used as a transport layer, and their characteristics were measured and analyzed. The value of the quantum efficiency and the values of the mobility of charge carriers in thin polyindole films were estimated.

## 1. Introduction

The achievements in the polymer chemistry and nanotechnology stimulate the intense development of organic electronics. The history of this scientific direction began in 1977, when the chemists Higger, McDiarmid and Shirakawa showed that polyacetylene modified with halogens can conduct electric current almost as well as a metal [1]. This discovery and other fundamental studies in the field of organic polymers contributed to the development of organic electronics that combines the developments in solid-state and molecular physics, organic and inorganic chemistry, electronics and printing. Sustained interest is naturally directed towards polyfunctional compounds that exhibit electrically conductive and optoelectronic properties. Electrically conductive polymers with a conjugated bond system, such as polythiophenes, polyphenylenevinylenes, polyanilines, etc. are most popular and widely studied for practical applications in organic electronics [2,3,4,5,6]. Polyindole and its derivatives stand out among the compounds mentioned above due to their photophysical properties along with high thermal and chemical stability, as well as complexation capability [7,8]. Varying the structures of polyindole derivatives makes it possible to improve their physicochemical properties and optimize the production method. At present, there are a lot of polyindole derivatives with customizable properties such as electrical conductivity, photoluminescence, and redox activity. Moreover, there are many reports on the synthesis of new composites and copolymers based on polyindoles, which further expand the applications of polyindole materials [9,10].

Materials based on conjugated electrically conductive polymers and inorganic substrates [11] are considered the most promising among the compounds used in this area. Various polyindoles, which can be responsible for optical conductivity, structural flexibility, and controlled electronic properties, are promising components for such materials [12]. The advantage of this series of polymers for optoelectronic applications is the presence of properties such as low energy optical transitions, extensive electrical conductivity, high electron affinity, and low ionization potentials [13]. It should be added that polyindoles have increasingly been used lately in organic electronics due to their unique electrophysical and photoluminescent properties, as well as because of the simplification of their synthesis, availability and low cost of raw materials, and stability in air [14,15,16]. The new forms of polyindoles synthesized recently [17] with their inherent photoluminescence and photoconductivity properties can find application in various optoelectronic devices. The purpose of this work was to study the optoelectronic properties of thin films of a new polyindole derivative in order to show that they can be used in photoresistors and phototransistors.

## 2. Materials and Methods

**Figure 1:**
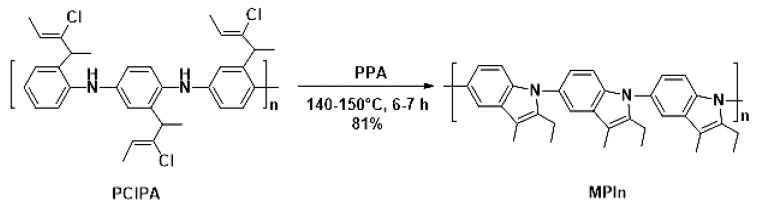
Synthesis of MPIn.

Poly(2-ethyl-3-methylindole) (MPIn) was obtained by intramolecular cyclization of poly(2-(2-chloro-1-methylbut-2-en-1-yl)aniline) performed by heating at 140–150 °C for 6–7 h in polyphosphoric acid (PPA), as described previously [17] (Figure 1). The MPIn yield was 81%. It should be noted that this reaction is a way to synthesize a new type of polyindole from a highly soluble polyaniline (PANI) derivative by polymer-analogous conversion. The polymer chain of MPIn involves a nitrogen atom, which undoubtedly has a significant effect on the physicochemical properties [17]. 

A 2600 Shimadzu spectrophotometer (Shimadzu Corporation, Kyoto, Japan) and an RF-5301 PC Shimadzu spectrofluorophotometer (Shimadzu Corporation, Kyoto, Japan) with 150 W Xenon lamp were used to record UV and fluorescence spectra at ambient temperature. The excitation wavelength was 440 nm. The polymer was dissolved in dimethyl sulfoxide (DMSO) to achieve a concentration of 10 mg L^−1^.

**Figure 2:**
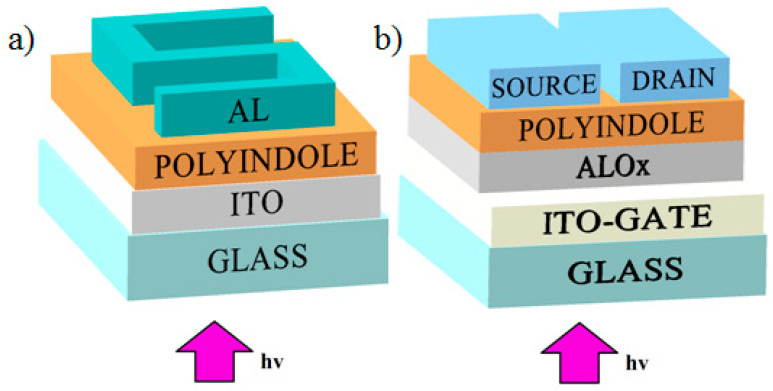
The structure of (**a**) a photoresistor and (**b**) phototransistor.

Samples of photoresistor devices were produced as follows. A glass substrate with a conductive indium tin oxide (ITO) coating was washed in an ethanol solution followed by drying in a muffle furnace for 10 min at 50 °C. On top of the ITO coating, a MPIn film was deposited by spin-coating from a solution. The rotation time was 120 s and the rotation speed for three samples was 700, 800 and 900 rpm. In this case, the resulting films were found to be 0.7, 0.5 and 0.4 µm thick, respectively. After that, the samples were placed in a muffle furnace at a temperature of 70 °C and kept there for half an hour to eliminate the remaining solvent. On top of MPIn, the upper aluminum electrode was deposited by thermal evaporation in a vacuum setup. The electrode had the form of an S-shaped track 1 mm wide (Figure 2a). The thickness of the aluminum electrode was 500 nm.

Samples of phototransistors based on polyindole films with a structure shown in Figure 2b were also created. Glass coated with a conducting layer of indium tin oxide (ITO) as a gate was used as a substrate. Before creating the required films, the substrates were annealed in a muffle furnace at 350 °C. AlO_X_ films 400 nm thick were created as the dielectric. AlO_X_ films were created by a wet method by centrifugation from solutions, the first two films from a solution with 1× concentration of AlO_X_ at 2000 rpm for 30 s, followed by annealing in a muffle furnace for 2 h at 350 °C of each film separately. Then two films from a solution with 5× concentration were obtained at 3000 rpm for 30 s with the same annealing. The polyindole film was created by centrifugation from a solution. The centrifugation parameters were 800 rpm, the rotation time was 2 min. The residual solvent was removed by heating in a muffle furnace for 30 min at 70 °C. Then, using thermal spraying in a VUP-5 vacuum system (residual pressure 2 × 10^−5^ mbar), (SELMI, Sumy, Ukraine), two aluminum electrodes (source and drain) with a thickness of 500 nm were applied. The gap between the contacts was 50 µm and their length was 2 mm. The thickness and structure of the film were determined using a Nanoeducator II atomic force microscope (NT-MDT, Moscow, Russia). 

The following devices were used in this study: a Mastech HY3005D-2 power supply (Precision Mastech Enterprises Company, Hong Hong, China), a GDM-8245 multimeter/ammeter (Good Will Instruments, Xinbei City, Taiwan), and a Hamamatsu LC8 light source (Hamamatsu Photonics, Shizuoka, Japan).

## 3. Results and Discussion

**Figure 3:**
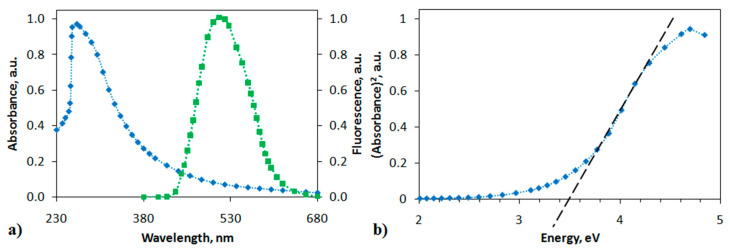
(**a**) Absorption (blue curve) and photoluminescence (green curve) spectra of MPIn, (**b**) dependence of α2 on photon energy hν.

The polymer-analogous reaction of the PANI derivative resulted in a change in the structure of the polymer chain. The formation of a polyindole moiety in the polymer chain increased the rigidity of the structure, which affected the physicochemical properties of MPIn. The absorption and photoluminescence spectra of the polyindole in DMSO (10 mg L^−1^) are shown in Figure 3. The electronic spectrum of MPIn reveals a single absorption maximum at 269 nm, which is characteristic of the entire series of polyindoles.

The emission spectra in Figure 3 indicate that the fluorescence peak of the MPIn under study are centered around 520 nm. Literature sources report the photoluminescence of various polyindole derivatives in the range of 430–470 nm [18,19]. However, it is observed that synthesized MPIn demonstrates a bathochromic shift relative to the previously studied polyindoles of a different type. It is also known, that polyindoles possess a photoluminescence due to their conjugated structure and, as consequence, a formation of indole fragment in the polyaniline chain leads to an increase in MPIn emission intensity. It is proposed that the observation of MPIn photoluminescence bathochromic shift is associated with changing the polymer structure due to 1,5-connection of monomer units. Moreover, formation of such indole structure in the polyaniline may cause an electronic effect on the transfer of an electron along the polyindole chain, that reveals as a bathochromic shift in the emission spectra. Additionally, it can be assumed that the shift and growth of MPIn emission associate with increase in inflexibility of MPIn structure in relation to previously studied polyindoles [17].

The absorption spectrum can also be used to estimate the band gap under the assumption that, most likely, interband direct allowed transitions occur, which are described by the formula [16]:

(1) α=A(hv−Eg)12

where *α* is the absorption coefficient, *A* is a constant, *hν* is the photon energy. Then it follows from the graph shown in Figure 3b that the band gap *E_g_* should be 3.5 eV. This value is consistent with the *E_g_* value previously obtained using a quantum-chemical calculation of MPIn [17].

The presence of photoluminescence implies that observation and photoconductivity are possible. Photoconductivity is an increase in the electrical conductivity of a semiconductor on exposure to light. A photoresistor and a phototransistor are the main devices whose operation is based on photoconductivity variations. To study the photoconductivity, current-voltage characteristics were measured under ultraviolet radiation at a radiation wavelength of 350 nm.

The dark current of polyindole films is of the order of 1 nA. Under UV irradiation, the photocurrent value is by three orders of magnitude greater than the dark value. The energy of UV quanta is 3.4 eV, which is comparable to the band gap. This causes the formation of electron-hole pairs, while the conductivity of MPIn thin films increases. 

**Figure 4:**
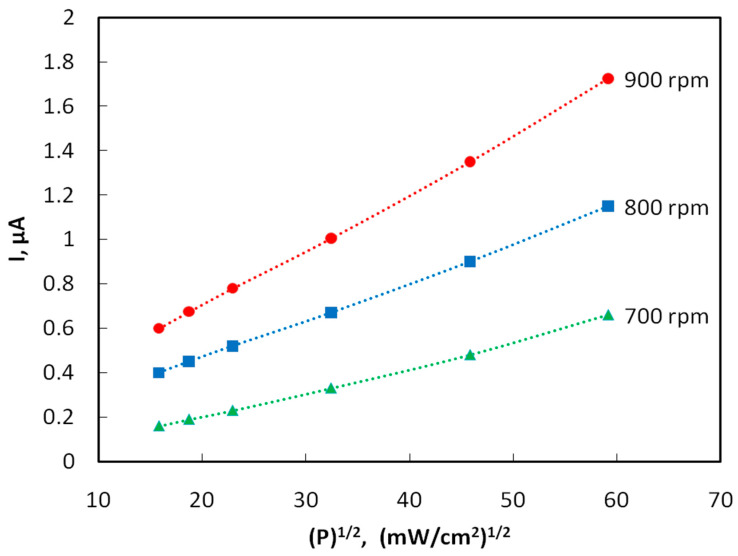
Plots of the photocurrent vs. the square root of the radiation power density for films obtained at different speeds of spin coating. The voltage between the electrodes is U = 10 V.

Figure 4 shows the plots of the photocurrent of samples obtained by spin coating at rotation speeds of 700, 800 and 900 rpm vs. the square root of radiation power density. The photoconductivity decreases 3–4 fold with an increase in the distance from the light source to the sample from 10 to 30 mm. In the case of interband quadratic recombination, the steady-state concentration of carriers generated by light, as well as the photoconductivity and photocurrent, obey a square-root dependence on the intensity of the light flux P [18]. Indeed, it turned out that this kind of dependence is observed experimentally.

The quantum efficiency η was estimated by the formulas: η = n/N, n = I/e, N = W/(hc/λ), W = q⸱s, where n is the number of photoelectrons arising per unit time, N is the number of photons incident on the surface of the photoresistor per unit time, I is the magnitude of the photocurrent, e is the electron charge, W is the radiation energy incident on the entire area of the photoresistor, s = 18 × 10^−6^ m^2^, per unit time; q = 35,000⸱W/m^2^ is the radiation flux density, and λ = 350 nm is the radiation wavelength. For example, let us calculate the quantum efficiency of samples obtained by centrifugation at a speed of 800 rpm (I = 1.15 × 10^−6^ A, n = I/e = 1.15 × 10^−6^ A/1.6 × 10^−19^ A/s = 0.719 × 1013 s^−1^, W = q × S = 35,000 W/m^2^ × 18 × 10^−6^ m^2^ = 1.94 × 10^−3^ J·s^−1^, N = W/(hc/λ) = 1.94 × 10^−3^ × 350 × 10^−9^ m/6.63 × 10^−34^ J·s × 3 × 10^8^ m × s^−1^ = 3.42 × 10^15^ s^−1^, η = n/N = 0.719 × 10^13^ s^−1^/3.4 × 10^15^ s^−1^ = 0.0021). The quantum efficiency η is found to be of the order of 10^−3^, i.e., slightly higher than that of naphthalene [19].

**Figure 5:**
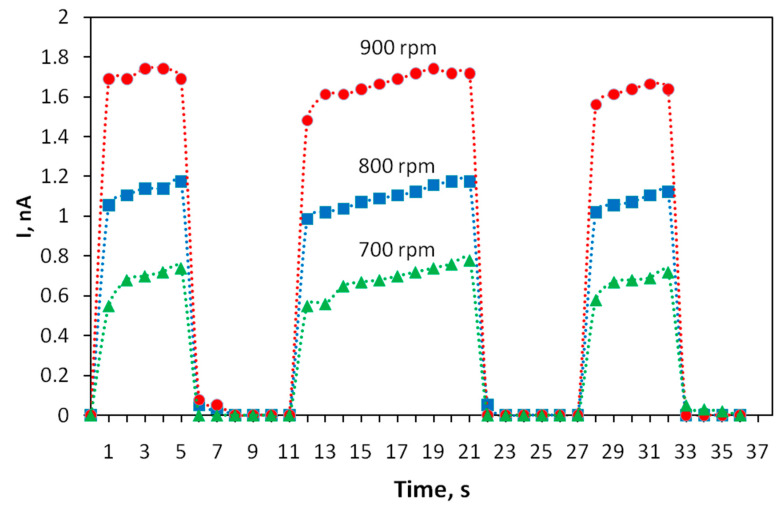
Kinetics of the photoresponse of the current through polyindole films obtained at different spin coating speeds. The voltage between the electrodes is U = 10 V.

The kinetics of the photoresponse of thin-film structures based on the polyindole was studied. To determine it, the variation in photoconductivity was measured upon applying a sequence of rectangular light pulses with durations of 5, 10 and 5 s. The results are shown in Figure 5. It turned out that the photoresponse of the transistor tracks the shapes of light pulses well and the rise and fall times of the photocurrent pulse do not exceed 1 s. A shorter time is typical of structures obtained at high speeds of rotation during spin coating.

**Figure 6:**
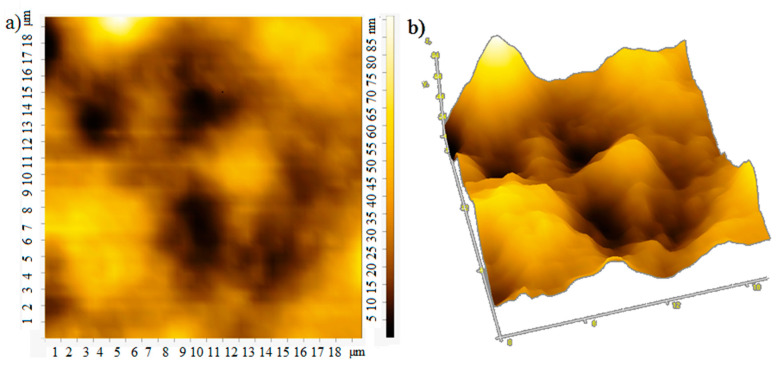

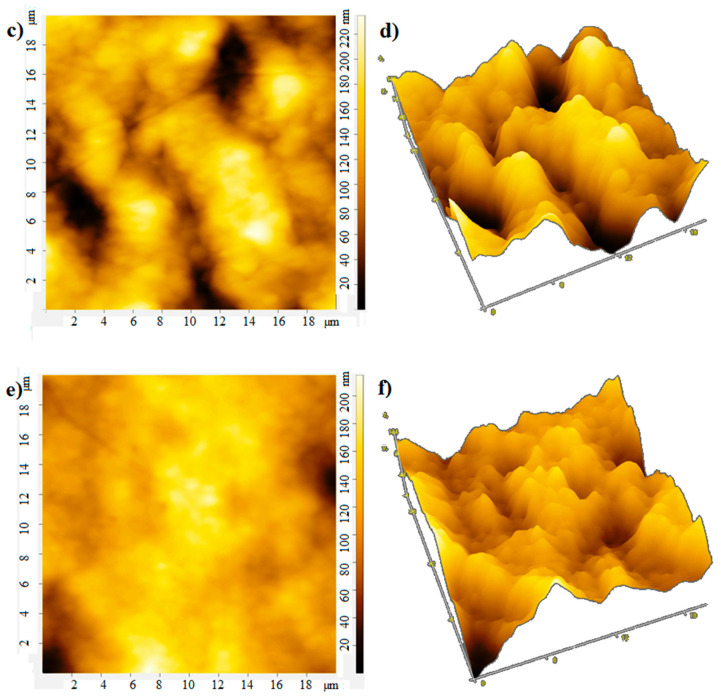
AFM: (**a**) 2D and (**b**) 3D image at rotation speed 700 rpm; (**c**) 2D and (**d**) 3D image at rotation speed 800 rpm; (**e**) 2D and (**f**) 3D image at rotation speed 900 rpm.

The film thickness was monitored using an atomic force microscope. The atomic force microscopy (AFM) images of the film surface are shown in Figure 6. The root-mean-square roughness of over the area of films obtained at rotation speeds of 700, 800, 900 rpm is 18, 25, and 13 nm, respectively. With an increase in the rotation speed, the uniformity of the film improves, the surface roughness decreases, which apparently leads to an increase in mobility of charge carriers [20], the photocurrent will also grow. In the case of large roughness, the “shading” effect can also manifest itself. The effective surface area on which the photons fall is smaller at low rotation speeds, which leads to a decrease in the photocurrent (Figure 4 and Figure 5).

**Figure 7:**
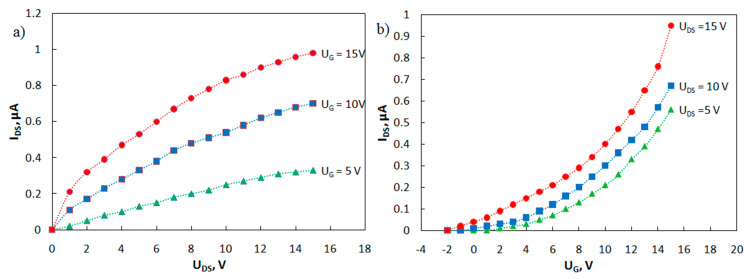
Family of (**a**) output and (**b**) transfer current-voltage characteristics of the phototransistor.

Families of output and transfer I-V curves of the phototransistor have been measured (Figure 7). A study on the current-voltage characteristics of the manufactured transistors showed that, in the absence of irradiation, the currents in the phototransistors are about 1 nA or less. Irradiation of the transistor gap regions with ultraviolet light (350 nm) increases the drain-source current by three orders of magnitude.

The carrier mobilities in the active layer *μ* of the fabricated OFETs were estimated using Formula (2):

*I_DS_* = (*W*/*L*)*μC*(*U_G_* − *U*_th_)*U_DS_* (2)

where *W* is the channel width, *L* is the channel length, *C* is the capacitance per square area of the gate dielectric AlO_X_ (for a thickness of 400 nm, *C* = 8.9 nF cm^−2^), *U*_G_ is the gate voltage, *U_DS_* is the drain-to-source voltage, and *U*_th_ is the threshold voltage. The estimated value of the carrier mobility is *μ* (MPIn) = 0.016 cm^2^ V^–1^ s^–1^, which is comparable to mobility for this class of compounds [21].

## 4. Conclusions

The results obtained are related to the photoconductivity of new polyindole derivative thin films. The absorption and photoluminescence spectra were measured, and it was found that synthesized MPIn demonstrates a bathochromic shift relative to the previously studied polyindoles of a different type. The photoconductive properties of polyindole thin films are investigated depending on the preparation conditions and surface morphology. Thin-film phototransistors based on thin MPIn films have been fabricated, and such characteristics as quantum efficiency and carrier mobility have been evaluated. The investigated form of polyindole is highly soluble, so the production of electronic components is compatible with the modern technology of printed organic electronics. All the measurements were carried out in atmospheric air, which is an advantage of the thin-film structures studied, since the majority of experimental devices based on other organic compounds can only operate in a chamber with an inert gas or dry nitrogen.

## Data Availability

Not applicable.
